# Prevalence and Characterization of *Staphylococcus aureus* Isolated From Retail Vegetables in China

**DOI:** 10.3389/fmicb.2018.01263

**Published:** 2018-06-14

**Authors:** Shi Wu, Jiahui Huang, Qingping Wu, Feng Zhang, Jumei Zhang, Tao Lei, Moutong Chen, Yu Ding, Liang Xue

**Affiliations:** ^1^State Key Laboratory of Applied Microbiology Southern China, Guangdong Institute of Microbiology, Guangzhou, China; ^2^Guangdong Provincial Key Laboratory of Microbial Culture Collection and Application, Guangdong Open Laboratory of Applied Microbiology, Guangzhou, China

**Keywords:** *S. aureus*, vegetable, antimicrobial resistance, enterotoxin, MLST, *spa* typing

## Abstract

*Staphylococcus aureu*s is a pathogen associated with serious community and hospital-acquired diseases. The aim of this study was to investigate the prevalence of *S. aureus* from retail vegetables in China and then characterized *S. aureus* isolates by antibiotic resistance, staphylococcal enterotoxin genes*, spa*-typing and multi-locus sequence typing. Of 419 retail vegetable samples from 39 cities in China during 2011–2016, 24 (5.73%) samples were positive for *S. aureus* and the geometric mean was 3.85 MPN/g. The prevalence of *S. aureus* was highest in lettuce (13/84, 15.48%) followed by tomato (7/110, 6.36%), caraway (2/87, 2.30%), and cucumber (2/128, 1.56%), whereas other vegetables were free of *S. aureus*. A total of 30 isolates were analyzed. For antibiotics susceptibility test, most isolates (93.3%) were resistant to ampicillin and penicillin, whereas all isolates were susceptible to linezolid, trimethoprim/sulphamethoxazole 1:19, nitrofurantoin, rifampicin, and teicoplanin. All isolates (30/30, 100%) were resistant or intermediate resistant to more than three tested antibiotics, including 9 isolates (30%) were resisted more than 10 antibiotics. Five isolates were resistant to cefoxitin and carried *mecA* genes which confirmed as MRSA. Of the 18 investigated SE genes, the *sem* gene was the most frequently detected (86.7%) followed by the *sec* (83.3%), *sep* (70.0%), *seg* (56.7%), *sel* (53.3%), *seh* (50.0%), *seq* (50.0%), *sej* (46.7%), *seb* (36.7%), *sen* (36.7%), and *ser* (33.3%) genes were harbored by more than one third of the isolates, whereas the *seo* and *seu* were detected in only 6.75% of the isolates. MLST and *spa* typing observed high genetic diversity in *S. aureus* isolated from retail vegetable in China. ST59-t437 was the predominant types (3/5, 60%) of MRSA isolates, whereas ST188-t189 was the predominant types (7/25, 28%) of MSSA isolates. Our study reflects that the retail vegetable in China could be contaminated with *S. aureus* but the levels of *S. aureus* were not very excessive. In addition, these isolates had virulence potential, most of them were enterotoxigenic and multiple antimicrobial resistance, should be draw public attention. These data have signification implications for epidemiological and public health studies of this pathogen.

## Introduction

*Staphylococcus aureus* is one of the most important pathogens and is responsible for various infections, such as wound infections and toxins-mediated syndromes as well as systemic and life-threatening diseases (Chambers and Deleo, 2009; Papadopoulos et al., 2018). Despite the ubiquitous distribution of *S. aureus* in nature, foods are still the important source of infection. Approximately 241,000 illnesses per year were causing foodborne disease in the United States by *S. aureus* (Scallan et al., 2011) and caused ~20–25% of foodborne bacterial outbreaks by *S. aureus* in China (Wang et al., 2014).

Differentiation between virulent and non-virulent strains is significant for evaluating the potential implications of the presence of this microorganism for food safety and public health. In general, *S. aureus* produced several virulence factors such as staphylococcal enterotoxins (SEs), leukocidin, exfoliatin, haemolysin, toxic shock syndrome toxin 1 (TSST-1) that can contribute in different ways to their pathogenicity. In which, SEs are heat stable proteins that are mainly associated with food poisoning outbreaks. It is shown that about 95% of staphylococcal food poisoning outbreaks were caused by the classical SE (SEA~SEE), and the remaining 55 of outbreaks were associated with other identified SEs (Altarazi et al., 2009). Furthermore, *S. aureus* also can be capable of acquiring antibiotic resistance determinants and exhibit resistance to multiple classes of antimicrobial agents. Methicillin-resistant *S. aureus* (MRSA) is practically resistant to all available β-lactam antimicrobial drugs. Nowadays, MRSA has been recognized as major cause of healthcare-associated infections worldwide and has been identified as an emerging pathogen outside the healthcare environment (Boucher, 2008).

Vegetables are essential to the human diet. Although the presence of *S. aureus* in retail food have been reported previously in China (Chao et al., 2015; Song et al., 2015; Yang et al., 2016a), very few studies focused on the retail vegetables, especial some types of vegetables, such as tomato, cucumber, lettuce, caraway which consumed raw popularly. Furthermore, the levels of contamination varied and influenced by food category, as well as geographical differences. It is necessary to investigate the qualitative and quantitative data of this bacterium in China to implement a system monitoring the prevalence patterns of *S. aureus* in food and environmental sources from different areas. Therefore, the aim of the present study was to investigate the prevalence and levels of *S. aureus* in retail vegetables from South China to North China and characterize these *S. aureus* isolates according to their antibiotic susceptibility profiles, enterotoxin genes, *spa* types and MLST types to determine their genetic background in China.

## Materials and methods

### Sample collection

**Figure 1:**
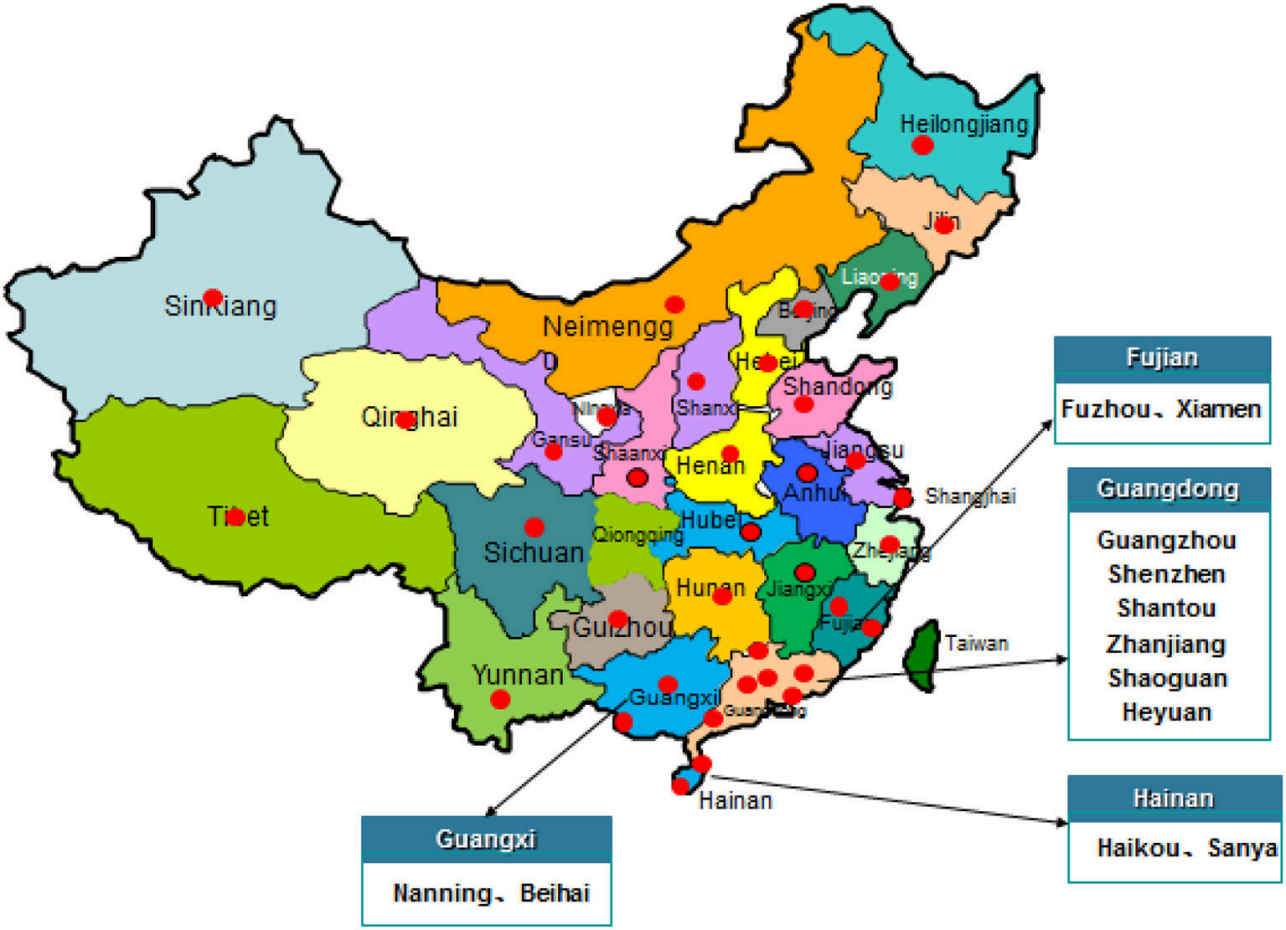
The locations of the sampling sites for this study in China.

Between July 2011 and June 2016, a total of 419 retail vegetable samples including 110 tomatoes, 128 cucumbers, 84 lettuces, 87 caraway and 10 other vegetables were collected from supermarkets, fairs and farmers' markets from 39 cities in 29 provinces and two directly controlled municipalities in China (Figure 1). The samples were placed in a cold box at a temperature approximately 4°C, tightly sealed with sterile plastic wrap, and transported to an accredited laboratory and subjected to microbiological analysis within 24 h.

### Isolation and identification of *S. aureus*

The isolation of *S. aureus* in the samples was determined using the most probable number (MPN) method according to GB 4789.10-2010 for food microbiological examination of *S. aureus* (National Food Safety Standards of China). Approximately 25 g of food sample was added to 225 mL of saline solution (Huankai, Guangzhou, China) for homogenization, then 1, 0.1, and 0.01 mL of each sample was inoculated in triplicate with trypticase soy broth (Huankai) supplemented with 10% NaCl and incubated at 37°C for 48 h, respectively. Loopfuls of the resulting cultures were streaked onto chromogenic *S. aureus* agar plates (Huankai), then incubated at 37°C for 24 h. Each positive sample selected 2–3 colonies with a pink color. After purification for 24 h at 37°C on NA plate (nutrient agar medium). Putative *S. aureus* isolates were tested for coagulase activity test by freeze-dried Rabbit Plasma (Huankai), and further confirmed by API STAPH identification test strips (bio Merieux, Marcy-1'Etoile, France) according to the manufacturer's instructions. The MPN value was determined on the basis of the number of positive tube(s) in each of the three sets using the MPN table.

### Antibiotic susceptibility testing

All confirmed *S. aureus* were tested for antibiotic susceptibility. It was using the Kirby–Bauer method (Bauer et al., 1966) which performed by standard disk diffusion on Mueller–Hinton agar incubated at 37°C for 24 h, following the guidelines of the Clinical and Laboratory Standards Institute (The Clinical and Laboratory Standards Institute, 2015). A total of 24 antibiotics (Oxoid, Basingstoke, UK) were classified into 14 different groups according to the WHO (Organization, 2007): amoxycillin/clavulanic acid (AMC, 30 μg), ampicillin (AMP, 10 μg), cefepime (FEP, 10 μg), Cefoxitin (FOX, 30 μg), penicillin G (P, 10U), ceftazidime (CAZ, 30 μg), amikacin (AK, 30 μg), gentamicin (CN, 10 μg), kanamycin (K, 30 μg), streptomycin (S, 25 μg), chloramphenicol (C, 30 μg), clindamycin (DA, 2 μg), erythromycin (E, 15 μg), telithromycin (TEL, 15 μg), ciprofloxacin (CIP, 5 μg), Norfloxacin (NOR, 10 μg), tetracycline (TE, 30 μg), Linezolid (LZD, 30 μg), rifampicin (RD, 5 μg), Trimethoprim/sulphamethoxazole 1:19 (SXT, 25 μg), Quinupristin/dalfopristin (QD, 15 μg), Teicoplanin(TEC, 30 μg), Nitrofurantoin (F, 300 μg) and Fusidic acid (FD, 10 μg). *Staphylococcus aureus* ATCC25923 and *Escherichia coli* ATCC25922 were included for quality control (The Clinical and Laboratory Standards Institute, 2015). The presence of the *mecA/mecC* gene was studied by PCR in all cefoxitin resistant isolates (Pérez-Roth et al., 2001; Stegger et al., 2012).

### Detection of staphylococcal enterotoxin genes

All isolates were tested by PCR for the presence of 18 genes coding for staphylococcal enterotoxins (*sea, seb, sec, sed, see, seg, seh, sei, sej, sek, sel, sem, sen, seo, sep, seq, ser*, and *seu*) (Varshney et al., 2009). The amplicons were subjected to electrophoresed on 1.5% agarose containing Goldview for 0.5 h at 120 V and visualized under a UV transilluminator gel imaging system (GE Healthcare, WI, USA). The images were saved as TIFF files for analysis.

### *spa*-typing

Sequence typing of the *S. aureus* protein A (spa) repeat region was amplified according to a published protocol (Shopsin et al., 1999). All isolates were analyzed using the primers *spa*-1113f (5′-TAAAGACGATCCTTCGGTGAGC-3′) and *spa*-1514r (5′-CAGCAGTAGTGCCGTTTGCTT-3′). The PCR amplification conditions were as follow: an initial cycle of 80°C for 5 min; 35 cycles of 94°C for 45 s, 60°C for 45 s, 72°C for 2 min and a final extension at 72°C for 10 min. The *spa* types were randomly assigned using the Spa Server website (http://spaserver2.ridom.de).

### Multi locus sequencing typing

The MLST scheme used to characterize *S. aureus* isolates is based on the sequence analysis of the following seven housekeeping genes: *arcC* (Carbamate kinase), *aroE* (Shikimate dehydrogenase), *glpF* (Glycerol kinase), *gmk* (Guanylate kinase), *pta* (Phosphate acetyltransferase), *tpi* (Triosephosphate isomerase), and *yqil* (Acetyle coenzyme A acetyltransferase) (Enright et al., 2013). The PCR amplification conditions were as follow: an initial cycle of 94°C for 5 min; 35 cycles of 94°C for 30 s, 55°C for 30 s, 72°C for 2 min and a final extension at 72°C for 10 min. The DNA fragments were purified by using a PCR purification kit (Qiagen, Genmany) and sequenced in each direction with Big Dye fluorescent terminators on an ABI 3730XL sequencer (Applied BioSystems). For each MLST locus, an allele number was given to each distinct sequence variant, and a distinct sequence type (ST) number was attributed to each distinct combination of alleles at the seven genes. Sequence types (STs) were determined by using the Staphylococcus aureus MLST database (https://pubmlst.org/saureus/). Sequence Type Analysis and Recombinational Tests software (S.T.A.R.T. ver.2; http://pubmlst.org/software/analysis/start2) was used to analyze the data of MLST.

### Statistical analysis

The bacterial numbers were converted to base-10 logarithms for statistical analysis. MPN values < 0.3 MPN/g were set to 0.15, and MPN values > 110 MPN/g were assigned the maximum value for this test (Motes et al., 1998). The chi-square test was used to determine differences in the prevalence and levels of *S. aureus*-positive samples between qualitative variables. All statistical analyses were performed using the SPSS v21.0 software package.

## Results

### Prevalence of *S. aureus* in retail vegetables in china

The results of the prevalence testing are summarized in Table 1. Overall, among 419 retail vegetables from 39 cities were examined, *S. aureus* were detected in 24 (5.73%) of samples from 20 cities and the geometric mean was 3.85 MPN/g. The prevalence of *S. aureus* was most common in lettuce (13/84, 15.48%) followed by tomato (7/110, 6.36%), caraway (2/87, 2.30%), and cucumber (2/128, 1.56%), whereas other vegetables (0/10, 0%) were free of *S. aureus*. Most of positive samples (91.67%) were less than 10 MPN/g by quantitative method, whereas none sample were reached 100 MPN/g.

**Table 1:** Prevalence and levels of *Staphylococcus aureus* in different retail vegetables.

**Types of product**	**NO. (%) of positives samples for *S. aureus***	**Strain Number of *S. aureus* isolates**	**Quantitative methods**	***S. aureus* level (MPN/g)**
			**MPN values (MPN/g)**	
Tomato	6.36 (7/110)	BJC2063	4.3	1.10
		CDC2563	0.36	
		XNC3013	0.3	
		SJZC3263	< 0.3	
		LSC3363	< 0.3	
		XGC3513	2.3	
		SYC3813	< 0.3	
Cucumber	1.56 (2/128)	ZCC65	12	7.15
		TYC2115	2.3	
Lettuce	15.48 (13/84)	SZN295	2.1	1.87
		ZJN395	2.3	
		HYC463	0.92	
		HYN497	0.74	
		STJ1741	4.3	
		SGC1813	4.3	
		HYC1863	0.3	
		HKC513	< 0.3	
		HKN545	< 0.3	
		SYN595	0.36	
		NNC663	3.5	
		SYJ3841	4.3	
		LSC4291	0.92	
Caraway	2.30 (2/87)	XGC3514	< 0.3	23.18
		CCJ4090	46	
Others	0 (0/10)	–	–	0.00
Total	5.73 (24/419)	–	–	3.85

### Antibiotic susceptibility testing

The antibiotic susceptibility results of 30 *S. aureus* isolates are showed in Table 2. Overall, the isolates were susceptible to LZD, SXT, F, RD and TEC, except one isolate having intermediate resistance to RD and 7 isolates having intermediate resistance to TEC. For 24 antibiotics, most isolates (93.3%) were resistant to AMP and P, followed by TE (43.3%), E (40.0%), K (33.3%), AMC (26.7%), S (23.3%), C (23.3%), DA (23.3%), CIP (23.3%), TEL (20%), and others (< 20%). However, all isolates were resistant or intermediate resistant to more than three tested antibiotics, of which 9 isolates (30%) were resistant to more than 10 antibiotics. Five isolates which were resistant to FOX and carried *mecA* genes, including 3 isolates collected from lettuce and 2 isolates obtained from caraway, confirmed as MRSA. These MRSA isolates showed resistant to all selected β-Lactams. Except that, there is no significantly difference between MRSA and MSSA for most antimicrobial tested.

**Table 2:** Results of antimicrobial susceptibility tests of *Staphylococcus aureus* isolates obtained from retail vegetables in China.

**Antimicrobial group**	**Antibiotics**	**Zone diameters (mm)**			***S. aureus*** **(*****n*** = **30)**		
		***R***	***I***	***S***	**NO.(%) of R**	**NO.(%) of I**	**NO.(%) of S**
β-Lactams	Amoxicillin/clavulanic acid (AMC)	≤19	–	≥20	8 (26.7)	–	22 (73.3)
	Ampicillin (AMP)	≤28	–	≥29	28 (93.3)	–	2 (6.7)
	Cefepime (FEP)	≤14	15–17	≥18	3 (10.0)	2 (6.7)	25 (83.3)
	Cefoxitin (FOX)	≤21	–	≥22	5 (16.7)	–	25 (83.3)
	Penicillin G (P)	≤28	–	≥29	28 (93.3)	–	2 (6.7)
	Ceftazidime (CAZ)	≤14	15–17	≥18	5 (16.7)	6 (20.0)	19 (63.3)
Aminoglycosides	Amikacin (AK)	≤14	15–16	≥17	1 (3.3)	6 (20.0)	23 (76.7)
	Gentamicin (CN)	≤12	13–14	≥15	5 (16.7)	0 (0.0)	25 (83.3)
	Kanamycin (K)	≤13	14–17	≥18	10 (33.3)	9 (30.0)	11 (36.7)
	Streptomycin (S)	≤11	12–14	≥15	7 (23.3)	18 (60.0)	5 (16.7)
Phenicols	Chloramphenicol (C)	≤17	18–20	≥21	7 (23.3)	8 (26.7)	15 (50.0)
Lincosamides	Clindamycin (DA)	≤14	15–20	≥21	7 (23.3)	3 (10.0)	20 (66.7)
Macrolides	Erythromycin (E)	≤13	14–22	≥23	12 (40.0)	2 (6.7)	16 (53.3)
	Telithromycin (TEL)	≤18	19–21	≥22	6 (20.0)	5 (16.7)	19 (63.3)
Fluoroquinolones	Ciprofloxacin (CIP)	≤15	16–20	≥21	7 (23.3)	3 (10.0)	20 66.7)
	Norfloxacin (NOR)	≤12	13–16	≥17	5 (16.7)	3 (10.0)	22 (73.3)
Tetracyclines	Tetracycline (TE)	≤14	15–18	≥19	13 (43.3)	0 (0.0)	17 (56.7)
Oxazolidinones	Linezolid (LZD)	≤20	–	≥21	0 (0.0)	–	30 (100)
Ansamycins	Rifampicin (RD)	≤16	17–19	≥20	0 (0.0)	1 (3.3)	29 96.7)
Sulfonamides	Trimethoprim/sulphamethoxazole 1:19 (SXT)	≤10	11–15	≥16	0 (0.0)	0 (0.0)	30 (100)
Quinolones	Quinupristin/dalfopristin (QD)	≤15	16–18	≥19	1 (3.3)	1 (3.3)	28 (93.3)
Glycopeptides	Teicoplanin (TEC)	≤10	11–13	≥14	0 (0.0)	7 (23.3)	23 (76.7)
Nitrofurantoins	Nitrofurantoin (F)	≤14	15–16	≥17	0 (0.0)	0 (0.0)	30 (100)
	Fusidic acid (FD)	≤24	–	≥25	3 (10.0)	–	27 (90.0)
Antimicrobial	1-5Antimicrobial				11		
	6-10Antimicrobial				10		
	11-15Antimicrobial				6		
	16-24Antimicrobial				3		

*R, resistant; I, intermediate resistance; S, susceptibility. The zone diameter was the standard from the CLSI of Staphylococcus spp*.

### Prevalence and distribution of enterotoxin genes

Among the 30 isolates analyzed, the isolates harbored at least one of the SE gene, including nine isolates carried more than 10 SE genes. Of the 18 investigated SE genes, the *sem* gene was the most frequently detected (86.7%) followed by the *sec* (83.3%), *sep* (70.0%), *seg* (56.7%), *sel* (53.3%), *seh* (50.0%), *seq* (50.0%), *sej* (46.7%), *seb* (36.7%), *sen* (36.7%), *ser* (33.3%), *sek* (30.0%), *sea* (26.7%), *sei* (23.3%), *sed* (10.0%), *see* (10.0%), *seo* (6.7%), and *seu* (6.7%) (Table 3). The classic SE genes (*sea, seb, sec, sed*, and *see*) showed 23.26% (50/215) of the detected genes, whereas the *egc* cluster (*seg, sei, sem, sen, seo*, and *seu*) accounted for 30.23% (65/215).

**Table 3:** Distributions of the 18 types of SE genes in vegetable *S. aureus* isolates in China.

**SE genes**		**No. (%) of positive isolates**				
	**Total**	**Tomato**	**Cucumber**	**Lettuce**	**Caraway**	
	**(*n* = 30)**	**(*n =* 8)**	**(*n* = 4)**	**(*n* = 15)**	**(*n* = 3)**	
Classic SE genes	*sea*	8 (26.7)	3 (37.5)	1 (25.0)	4 (26.7)	–
	*seb*	11 (36.7)	5 (62.5)	1 (25.0)	3 (20.0)	2 (66.7)
	*sec*	25 (83.3)	8 (100.0)	4 (100.0)	10 (66.7)	3 (100.0)
	*sed*	3 (10.0)	2 (25.0)	1 (25.0)	–	–
	*see*	3 (10.0)	2 (25.00)	1 (25.0)	–	–
Non-classic SE genes:egc cluster	*seg*	17 (56.7)	7 (87.5)	2 (50.0)	5 (33.3)	3 (100.0)
	*sei*	7 (23.3)	2 (25.0)	1 (25.0)	2 (13.3)	2 (66.7)
	*sem*	26 (86.7)	8 (100.0)	4 (100.0)	11 (73.3)	3 (100.0)
	*sen*	11 (36.7)	7 (87.5)	1 (25.0)	3 (20.0)	–
	*seo*	2 (6.7)	2 (25.0)	–	–	–
	*seu*	2 (6.7)	2 (25.0)	–	–	–
Non-classic SE genes: other SE genes	*seh*	15 (50.0)	4 (50.0)	4 (100.0)	7 (46.7)	–
	*sej*	14 (46.7)	8 (100.0)	2 (50.0)	1 (6.7)	3 (100.0)
	*sek*	9 (30.0)	4 (50.0)	–	3 (20.0)	2 (66.7)
	*sel*	16 (53.3)	7 (87.5)	4 (100.0)	5 (33.3)	–
	*seq*	15 (50.0)	5 (62.5)	3 (75.0)	4 (26.7)	3 (100.0)
	*sep*	21 (70.0)	8 (100.0)	4 (100.0)	7 (46.7)	2 (66.7)
	*ser*	10 (33.3)	6 (75.0)	1 (25.0)	–	3 (100.0)

### Molecular characterization of *S. aureus*

*Spa*-typing detected a total of 16 different types from 30 *S. aureus*, including one new type (t16985). The most common *spa* type was t189 (23.33%) followed by t091 (13.33%) and t437 (13.33%). Other *spa* types, such as t114, t1987, t2874, t10419, t2467, t701, t304, t377, t803, t3092, and t034, were both singletons independently of the isolates source (Table 4).

**Table 4:** Allelic profile of vegetable *S. aureus* isolates for MLST and *spa* typing.

**Method**	**Criterion**	**No. (%) of positive isolates**				
		**Total (*n* = 30)**	**Tomato (*n* = 8)**	**Cucumber (*n* = 4)**	**Lettuce (*n* = 15)**	**Caraway (*n* = 3)**
MLST	ST188	7 (23.3)	1 (3.3)	3 (10.0)	3 (10.0)	–
	ST1	5 (16.7)	2 (6.7)	–	3 (10.0)	–
	ST7	5 (16.7)	–	–	4 (13.3)	1 (3.3)
	ST59	4 (13.3)	1 (3.3)	–	1 (3.3)	2 (6.7)
	ST6	3 (10.0)	1 (3.3)	1 (3.3)	1 (3.3)	–
	ST72	1 (3.3)	1 (3.3)	–	–	–
	ST398	1 (3.3)	–	–	1 (3.3)	–
	ST20	1 (3.3)	–	–		–
	ST630	1 (3.3)	–	–	1 (3.3)	–
	ST15	1 (3.3)	1 (3.3)	–	1 (3.3)	–
	ST2196	1 (3.3)	1 (3.3)	–	–	–
*spa*	t189	7 (23.3)	1 (3.3)	3 (10.00)	3 (10.0)	–
	t091	4 (13.3)	–	–	3 (10.0)	1 (3.3)
	t127	3 (10.0)	2 (6.7)	–	1 (3.3)	–
	t437	4 (13.3)	1 (3.3)	–	1 (3.3)	2 (6.7)
	t114	1 (3.3)	–	–	1 (3.3)	–
	t16895	1 (3.3)	–	–	1 (3.3)	–
	t1987	1 (3.3)	1 (3.3)	–	–	–
	t2874	1 (3.3)	–	–	1 (3.3)	–
	t10419	1 (3.3)	1 (3.3)	–	–	–
	t2467	1 (3.3)	–	1 (3.3)	–	–
	t701	1 (3.3)	–	–	1 (3.3)	–
	t304	1 (3.3)	1 (3.3)	–		–
	t377	1 (3.3)	–	–	1 (3.3)	–
	t803	1 (3.3)	–	–	1 (3.3)	–
	t3092	1 (3.3)	1 (3.3)	–	–	–
	t034	1 (3.3)	–	–	1 (3.3)	–

**Figure 2:**
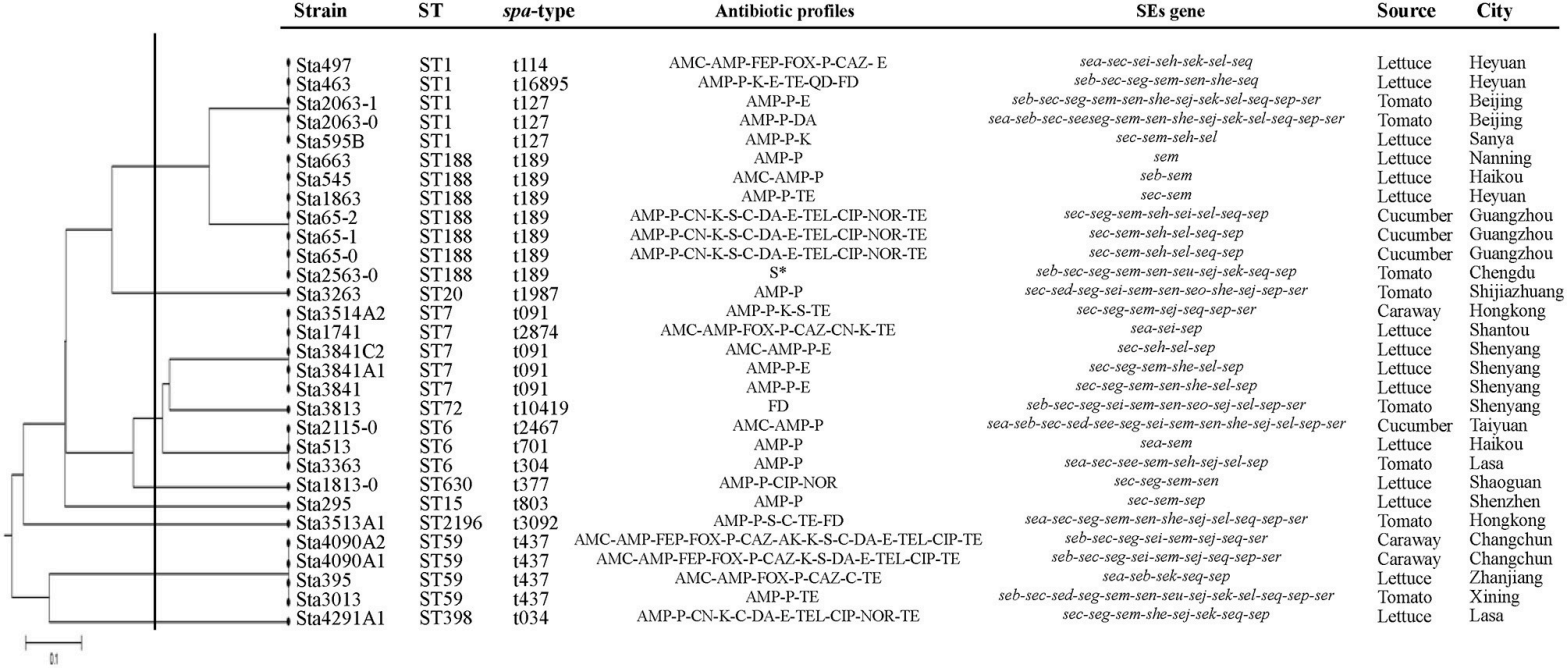
The UPGMA (unweighted pair group method with arithmetic mean) tree of the 7 multi-locus sequence typing loci of vegetable *S. aureus* isolates. S*, susceptible. This tree was generated using the S.T.A.R.T (version 2).

Eleven different STs were identified for all isolates from retail vegetables (Table 4). ST188 (7/30, 23.3%), ST1 (5/30, 16.7%), ST7 (5/30, 16.7%) and ST6 (3/30, 10%) were the frequent STs in this research. A phylogenetic tree based on the 7 concatenated MLST sequences (Figure 2) shows the relatedness between the vegetable isolates. Combining the STs and *spa* types, ST59-t437 was the predominant types (3/5, 60%) of MRSA isolates, whereas ST188-t189 was the predominant types (7/25, 28%) of MSSA isolates. Most isolates demonstrated high concordance between STs and *spa* types. Three ST6 isolates showed different *spa* types (ST6-t2467, ST6-t701, and ST6-t304) isolated from different places.

## Discussion

The presence of *S. aureus* in vegetable have been reported are very fewer than other types of food. Furthermore, full-scale geographic investigation is necessary. In this study, we investigate retail vegetables from 39 cities in China, covering most provincial capitals of China, which showed that 24 positive samples were detected *S. aureus* including 4 MRSA-positive samples from 419 retail vegetables in 20 cities. It indicated that the retail vegetable in China could be contaminated with *S. aureus*. In China, there is no standard limit of *S. aureus* in vegetable. In the present study, most of positive samples (91.67%) were less than 10 MPN/g and only one sample reached 46 MPN/g from caraway. Thus, refer to the limit of *S. aureus* in other types of food (GB 29921-2013), on the whole, levels of *S. aureus* in retail vegetable in China were not very excessive. Compare with other surveys, our result was lower than those from retail raw meat, infant formula milk, lettuces, or aquatic products in China, which reported that the contamination of *S. aureus* was 24.2, 11.2, 10.0, and 37.2%, respectively (Seo et al., 2010; Wang et al., 2012, 2013; Rong et al., 2017). However, vegetables are essential to the human diet in China, especially with the rationalization of diet structure recent years. Furthermore, the contamination focus on lettuce, tomato, caraway, and cucumber in this study. These types of samples always consumed raw, which indicated consumers may be exposed to infection, and should be draw public attention.

In recent years, the spread of antibiotic resistance among strains of *S. aureus* is of great public and clinical concern in the treatment of staphylococcal infections (Shankar, 2012). Many researchers have reported resistance strains of *S. aureus* isolates from various food samples in different countries (Argudín et al., 2012; Yang et al., 2016b; Wang et al., 2017). In the current study, 76.7% of vegetable *S. aureus* isolates (23/30) were resistant or intermediate resistant to more than three tested antibiotics classes, of which 9 isolates were resistant to more than 10 antibiotics. At present, our data is higher than previous reports among various food resources in other countries (Kozytska et al., 2010; Shahraz et al., 2012; Spanu et al., 2012; Ge et al., 2017; Papadopoulos et al., 2018). In this study, 93.3% of isolates were resistant to ampicillin and penicillin, showing lower than the research by Hong et al. (2015) from leaf vegetables (96.3%) in Korea. Resistance to other antimicrobials, e.g., tetracycline, erythromycin, gentamicin, and ciprofloxacin, is similar to the reports of Chao et al. (2007), who observed that 49.4% of *S. aureus* isolates from food products were resistant to tetracycline, 24.1% to erythromycin, and 13.8% to gentamicin. Considering these antibiotics have been increasingly used in animal breeding or human treatment and exchange of antibiotic-resistant genes by the mobile genetic elements (MGEs) (Lindsay, 2014), it is not surprising that resistant strains become more common in the present. As we know, methicillin-resistant *S. aureus* (MRSA) represents a serious public health issue due to its ability to colonize and infect humans and animals, there were 5 MRSA strains isolated from 4 positive samples in this study. Cross contamination from environments may be the major reasons because animal-derived food products are widely known to be an important reservoir for MRSA (Petinaki and Spiliopoulou, 2012). However, the high antimicrobial resistance of *S. aureus* observed in this study should receive much attention. Moreover, controlled use of antimicrobials would limit the emergence of drug-resistant bacteria.

Generally speaking, staphylococcal enterotoxins (SEs) encoded by SE genes, which play an important role in the pathogenicity of the bacteria. In our study, 18 SE genes were tested and all isolates harbored at least one of the SE genes. Three genes, *sem* (86.7%), *sec* (83.3%), and *seq* (70.0%) were more frequently detected, whereas *sed* (10.0%), *see* (10.0%), *seo* (6.7%), and *seu* (6.7%) were detected at lower frequencies. From these types of SEs, it was suggested that about 95% of staphylococcal food-poisoning outbreaks were caused by strains carrying the classical SE (SEA-SEE) (Mashouf et al., 2015). Chao et al. (2015) have analyzed different sources of SE gene distributions, which found that the classic genes in both foodborne isolates and human origin isolates were significant higher than that in animal origin. In this study, 23.3% of the classic SE genes were detected, which means potential virulence as these isolates and potentially capable of causing an epidemic. Furthermore, the *egc* cluster (*seg, sei, sem, sen, seo, seu*) was widely distribute in clinic isolates and as a putative nursery of enterotoxin genes (Jarraud et al., 2001). Previous studies have showed that strains carried novel SE or SE-like genes could cause SFPs (McLauchlin et al., 2000; Argudín et al., 2010; Johler et al., 2015), it found 30.2% of detected genes of the *egc* cluster, higher than the classical SE genes, which is consist with previous reports (Smyth et al., 2005). However, most of SEs genes are carried and disseminated through mobile genetic elements that their spread among *S. aureus* isolates can modify their ability to cause disease and contribute to the evolution of this important pathogen. So the isolates harboring these genes were potential hazards to food safety.

MLST and *spa* typing observed high genetic diversity in *S. aureus* isolated from retail vegetable in China. In this study, it detected 16 different *spa* types and 11 different STs by two typing methods from 30 *S. aureus* isolates. The major STs, such as ST1, ST188, ST7, and ST6 in this study, was consist with (Song et al., 2015)'s result but was distinguish with previous studies, e.g., ST8 in US retail meat, ST1 in Chinese RTE food and ST152 in South Italy dairy product (Basanisi et al., 2017; Ge et al., 2017; Yang et al., 2018). It is indicated the genetically diversity with geographic origins and types. However, virulence genes and antibiotic profiles remarkably variation in same types. For instance, sta663 and sta65-0 belonged to ST188 from different cities, of which sta663 harbored *sem* gene and resistant to 2 antibiotics, whereas sta65-2 harbored *sec-seg-sem-seh-sei-sel-seq-sep* and resist for 12 antibiotics.

As shown in Figure 2, we observed a good agreement between MLST and *spa* typing result. *Spa* typing is a useful genotyping method for *S. aureus*. Compared with MLST typing, *spa* typing method was more discrimination. Three ST6 isolates showed different *spa* types (ST6-t2467, ST6-t701, and ST6-t304) isolated from different places. It also could distinguish antibiotic resistance in this study, which showed difference between the MRSA isolates (ST1-t114 and ST7-t2874) and MSSA isolates (ST1-t127 and ST7-t091). Furthermore, ST59-t437, which showed the predominant types of MRSA isolates in this study, was also occurs in Hong Kong, China, Vietnam, Japan, and Australia for community-associated MRSA (CA-MRSA) (Coombs et al., 2006; Tang et al., 2007; Ho et al., 2008; Higuchi et al., 2010; Wu et al., 2010). In China, Wang et al. (2004) was first reported this type of CA-MRSA. They found that 16 of 17 CA-MRSA isolates from children admitted to hospital with skin and soft tissue infections (SSTIs) from 1997 to 2002 belonged to ST59, and carried PVL genes. After that, more and more researches reports ST59 CA-MRSA. According to Chuang and Huang (2013) ‘s review, ST59 (and its single locus variant ST338) was the major lineage accounting for up to two-thirds of isolates, followed by ST910-IVa-t318 and ST1 in CA-MRSA. Therefore, it should pay great attention for this situation which found ST59-t437 was the predominant types of MRSA isolates in retail vegetable in our study.

In summary, our study is the first systematical investigation of prevalence and contamination level for *S. aureus* isolated from retail vegetable in China. Our reports showed that the retail vegetable in China could be contaminated with *S. aureus* but the levels of *S. aureus* were not excessive. Most vegetable isolates exhibited resistance to different antimicrobials. In addition, different toxins genes existed in many *S. aureus* isolates. Thus, improved effective measures should be implemented in the food processing to control contamination and exposure to *S. aureus*. Besides, some MRSA strains, relevant to major CA-MRSA clone in Asia, were found in this study. Thus, it should be draw public attention and further studies are required to ascertain major clone of MRSA from retail food in China.

## Author contributions

SW, JH, and TL conceived and designed the experiments. JH and FZ performed the experiments. SW and JH analyzed the data. LX, MC, and YD contributed reagents, materials, and analysis tools. SW, QW, and JZ contributed to the writing of the manuscript.

### Conflict of interest statement

The authors declare that the research was conducted in the absence of any commercial or financial relationships that could be construed as a potential conflict of interest.
